# Comparison of Mirroring and Overlapping Analysis and Three-Dimensional Soft Tissue Spatial Angle Wireframe Template in Evaluating Facial Asymmetry

**DOI:** 10.3390/bioengineering12010079

**Published:** 2025-01-16

**Authors:** Gengchen Yang, Liang Lyu, Aonan Wen, Yijiao Zhao, Yong Wang, Jing Li, Huichun Yan, Mingjin Zhang, Yi Yu, Tingting Yu, Dawei Liu

**Affiliations:** 1 Department of Orthodontics, Peking University School and Hospital of Stomatology, National Center of Stomatology, Beijing 100081, China; 2211210565@bjmu.edu.cn (G.Y.); lvliang_1996@126.com (L.L.); lijingchn@foxmail.com (J.L.); 1911110509@pku.edu.cn (H.Y.); 1610303110@pku.edu.cn (M.Z.); yuyinaive@outlook.com (Y.Y.); tiffanyutt@126.com (T.Y.); 2 National Clinical Research Center for Oral Diseases, National Engineering Research Center of Oral Biomaterials and Digital Medical Devices, Beijing 100081, China; wan960608@163.com (A.W.); kqcadcs@bjmu.edu.cn (Y.Z.); kqcadc@bjmu.edu.cn (Y.W.); 3 Beijing Key Laboratory of Digital Stomatology, Beijing 100081, China; 4 Center of Digital Dentistry, Department of Prosthodontics, Peking University School and Hospital of Stomatology, National Center of Stomatology, Beijing 100081, China; 5 Research Center of Engineering and Technology for Computerized Dentistry Ministry of Health, Beijing 100081, China

**Keywords:** three-dimensional, wireframe template, facial asymmetry, aesthetics

## Abstract

Aim: The purpose of this study was to evaluate the accuracy and efficacy of a new wireframe template methodology in analyzing three-dimensional facial soft tissue asymmetry. Materials and methods: Three-dimensional facial soft tissue data were obtained for 24 patients. The wireframe template was established by identifying 34 facial landmarks and then forming a template on the face with the MeshLab 2020 software. The angle asymmetry index was automatically scored using the template. The mirroring and overlapping technique is accepted as the golden standard method to diagnose facial asymmetry by acquiring deviation values of one’s face. Consistency rates between the two methodologies were determined through a statistical comparison of the angle asymmetry index and deviation values. Results: Overall consistency rates in the labial, mandibular angle, cheek, chin, and articular regions were 87.5%, 95.8%, 87.5%, 91.7%, and 100%, respectively. Regions with consistency rates in three dimensions of more than 85% are the *x*-axis and the *z*-axis of all regions and the *y*-axis of the mandibular angle, chin, and articular region. Conclusions: Soft tissue facial asymmetry can be diagnosed accurately and effectively by using a three-dimensional soft tissue spatial angle wireframe template. Precise localization of asymmetry can be offered, and indiscernible tiny asymmetry can be identified.

## 1. Introduction

Facial asymmetry, often caused by skeletal or dental discrepancies, profoundly affects a patient’s appearance, self-esteem, and overall oral health [1,2]. An optimal diagnostic protocol not only augments the orthodontist’s proficiency in pinpointing and quantifying facial deviations but also facilitates the formulation of individualized therapeutic strategies that adeptly address the foundational etiologies.

Within the orthodontic area, numerous techniques have been employed to evaluate facial asymmetry. Traditionally, the reliance has been on two-dimensional (2D) modalities [3,4], with cephalometric radiography and frontal imagery serving as primary tools for such assessments. Nevertheless, these 2D techniques exhibit intrinsic limitations, especially in their inability to aptly capture the depth and intricacies of anatomical structures [5], like those of the maxillary and mandibular regions. This has prompted a shift towards three-dimensional (3D) diagnostic methods. A prime example of this evolution is the adoption of cone beam computed tomography (CBCT) [6,7], recognized for its enhanced accuracy in visualizing hard tissue structures. Despite its advantages, CBCT’s capacity to detail soft tissue structures remains suboptimal, necessitating further advancements. In this context, 3D facial scanning has been increasingly recognized and utilized by practitioners [8,9]. Its advantages, such as its non-invasiveness and superior depiction of 3D soft tissue nuances [10], make it a preferred choice, transcending the constraints observed in 2D techniques and CBCT.

Within orthodontics, the mirroring and overlapping method, underpinned by 3D facial scan insights, has become an esteemed evaluation tool, setting a benchmark for facial asymmetry analysis [11,12,13]. By computing facial side deviations, it offers a chromatographic representation, simplifying the task of spotting asymmetrical facial areas and calculating their intensity. However, its potential to occasionally downplay asymmetry’s magnitude and seriousness is evident. Additionally, while the Facial Asymmetry Index (FAI) [14,15] and statistical shape analysis [3,16,17] have been applied clinically, it lack universal endorsement due to the inherent challenges in determining consistent reference planes and pinpointing facial landmarks with precision. Therefore, further research is required to enhance the assessment of facial asymmetry.

Despite considerable research, there is still no widely accepted standard for evaluating facial asymmetry. Our previous study [18] introduced a novel method known as the “3D facial wireframe template”. This template automatically identifies facial soft tissue landmarks and creates a wireframe representation of the face by connecting line segments between these landmarks. Using this template improves the visual depiction of facial asymmetry and enhances its accuracy during evaluation. As a result, it enables clinicians to provide more precise clinical diagnoses and employ more effective treatment approaches. In this study, we compared the 3D facial wireframe template to the conventional golden standard method of the mirroring and overlapping analysis, in order to evaluate its accuracy and effectiveness in assessing facial asymmetry.

## 2. Materials and Methods

The study was carried out from March 2021 to March 2022. It was approved by the Biomedical Ethics Committee of the Peking University Stomatological Hospital (Approval No. PKUSSIRB-2021026208). The authors explained the relevant procedures to all participants and made sure they knew we would use their face scan data and that they would contribute to the research. All participants complied by signing an informed consent form.

All facial soft tissue data of these patients were measured by the mirroring and overlapping analysis and the wireframe analysis. Overall and three-dimensional recognition rates and consistency rates in five regions were calculated.

### 2.1. Data Collection

A non-inferiority test was conducted using PASS software (version 15.0, NCSS) based on the pre-test results. We obtained a sample size comprising >20 cases (power = 0.90, α = 0.05, and R_0_ = 0.67).

A total of 24 patients, including 14 males and 10 females, aged 12–32 years, who visited the Department of Orthodontics, Peking University Stomatological Hospital, for an initial consultation between March 2021 and March 2022 were randomly selected and included in the study.

The inclusion criteria were the following:

- Diagnosis of facial asymmetry by experienced orthodontists and verification by the mirroring overlapping analysis with maximum differences of more than 3 mm
- Presence of informed consent.
- The exclusion criteria were the following:
- History of craniofacial deformities;
- History of facial soft tissue trauma;
- Previous orthodontic treatments or orthognathic surgery.

The 3D facial scan data of the patients were acquired by using the Bellus 3D Arc 1 system (Bellus3D, Inc. Campbell, CA, USA). During scanning, the participants maintained a natural head position, eyes flat in front, and natural expression to prevent the obstruction of the facial contour area. All data were captured in the “OBJ” format.

### 2.2. Division of Regions of Interest and Data Processing

According to the clinical division of the face, the middle and lower parts of the face were divided into five regions of interest (the labial, mandibular angle, cheek, chin, and articular regions).

Obvious asymmetry can always be seen in the chin regions of patients with facial asymmetry in orthodontic clinical practice, and the findings of Dong T et al. [19] have also verified that chin asymmetry has a great influence on facial esthetics. Therefore, we took the chin regions as important regions of interest in the study.

Asymmetry of the labial and the cheek regions is often shown in asymmetric patients. Aoyama I et al. [20] found that surgical orthodontic patients with facial asymmetry frequently show asymmetry of the lips. Fan Y et al. [21] found that the lower cheek asymmetry was detected to have more extensive and of a greater magnitude of asymmetry than other facial anatomical regions. Therefore, the labial and cheek regions are also important regions of interest that should not be neglected.

There are also skeletal deformities in most asymmetric patients, and asymmetry of bilateral mandibular angles is a common cause of skeletal facial asymmetry, leading to asymmetry in the corresponding soft tissue areas. Khaghaninejad MS et al. [22] used the difference between two gonial angles to determine the amount of asymmetry in the study, which further confirmed the contribution of asymmetry of the mandibular angle regions to facial asymmetry. Moreover, patients with facial asymmetry often have associated joint issues. Inui M et al. [23] found that facial asymmetry due to mandibular lateral displacement was a relatively common problem in patients with temporomandibular joint disorders. So, we also paid special attention to asymmetry in the articular regions.

Definitions of the five regions are as follows:

The labial region: A rectangular region where the upper boundary is the nose floor, the lower boundary is the mentolabial groove, and the sides are bounded by two labiofacial grooves.

The mandibular angle region: A fan-shaped area where the upper boundary is the horizontal plane where the lowest point of the earlobe is located, the lower boundary is the vertical plane where the posterior edge of the zygomatic bone is located, and the semi-circular boundary is the curve along the shape of the mandibular angle.

The cheek region: The upper boundary is the lower edge of the zygomatic bone, the lower boundary is the lower edge of the mandibular, one side is bounded by the nasofacial groove, and another side is bounded by the boundary of the face in X, Y axis.

The chin region: The upper boundary is mentolabial groove, and the lower and the side boundaries are the same as those of mental protuberance.

The articular region: A rectangular region where the upper boundary is the horizontal plane where the highest point of the helix is located, the lower boundary is the horizontal plane where the lowest point of the earlobe is located, one side is bounded by the tragus, and another side is bounded by the posterior edge of the zygomatic bone.

Each spatial line angle metric represents the asymmetry of that site in a particular 3D direction. The details of the wireframe template are specifically shown in our published paper [18].

**Figure 1:**
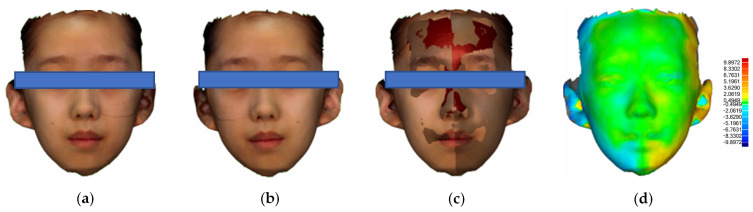
Schematic diagram of mirroring overlapping bias analysis: (**a**) Original face-scan image. (**b**) Mirrored face-scan image. (**c**) Aligned overlapped image. (**d**) Deviation colormap.

For the mirroring and overlapping analysis, data processing was performed using Geomagic Studio 2015 (Geomagic, Inc., Morrisville, NC, USA) and MeshLab (MeshLab 2020.12, 3D) based on the Frankfort Plane, with the upper boundary of the data retained at the hairline, the lower boundary at the angle of the neck, and the left and right boundaries covering the ears. Data mirroring was executed and synchronized with the primary data. Then best-fit alignment was executed on the original data, the mirroring data was based on the midline of the face, and deviation values representing facial asymmetry in five regions of interest across three dimensions were determined. Subsequently, a deviation colormap was developed to delineate the asymmetries across these regions. The entire procedure is illustrated in Figure 1.

**Figure 2:**
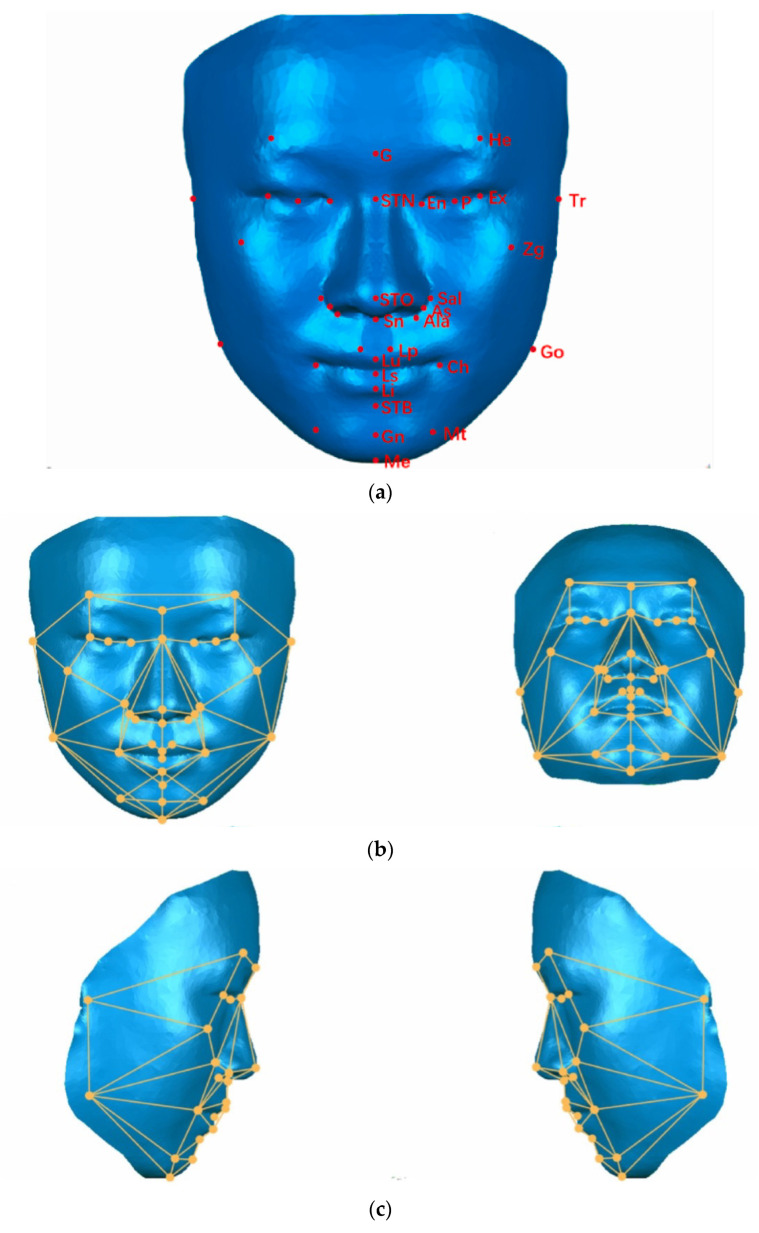
Schematic diagram of the reference for establishing the wireframe template. (**a**) A total of 34 soft tissue facial landmarks of the template. (**b**) The template on the front face. (**c**) The template on the lateral face.

For the wireframe template, 34 soft tissue landmarks (Figure 2) were automatically identified by the Procrustes Analysis (PA) algorithm software MATLAB R2019b (The MathWorks, Inc., Natick, MA, USA), Open-Source Nonrigid (OSN) software MATLAB R2019b, MATLAB R2019b, Open-Source Rigid Software (OSS) MATLAB R2019b, and OSN alignment program MeshMonk [24], and then were connected sequentially and constructed to form a wireframe template on the face. According to our previous study [18], the spatial line angle parameters in the template were selected as presented in Table 1. An angular asymmetry index for angle differences between the left and the right side was used by the wireframe templates to represent facial asymmetry across five regions of interest in three dimensions and was calculated with the following formula:

Angle asymmetry index (AAI) = |R−L|/L × 100%.(1)

**Figure 3:**
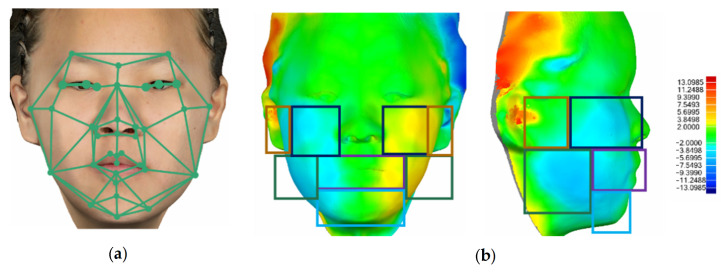
Schematic comparison of the wireframe template analysis and the mirroring and overlapping analysis for evaluating facial asymmetry. (**a**) Methods of the wireframe template analysis and the mirroring and overlapping analysis. (**b)** Deviation colormap of one patient with the mirroring and overlapping analysis.

The 3D facial wireframe template analysis and the mirroring and overlapping analysis of a patient are shown in Figure 3. For this patient, the most seriously asymmetrical areas were represented in red and blue, where an asymmetry of 12.194 mm existed. The most symmetrical areas were represented in green, where no or tiny asymmetry existed.

### 2.3. Consistency Test Methods and Criteria of Facial Asymmetry in the Mirroring and Overlapping Analysis and the Wireframe Template Analysis

All facial soft tissue data of these patients were measured by the mirroring and overlapping analysis and the wireframe analysis. These measurements were performed by a single author and repeated once under the same conditions after one week. Overall recognition rates and consistency rates, recognition rates, and consistency rates in five regions across three dimensions were calculated.

The unit of deviation values from the mirroring and overlapping analysis is a millimeter and that of the angle asymmetry index from the wireframe template is a percent. So, the values and indexes, which are continuous variables, were transformed into categorical variables. The degree of facial asymmetry like mild, moderate, and severe asymmetry in different criteria were used to test the consistency of the two methods with the outcomes from the mirroring and overlapping analysis as the benchmark.

Several research studies [3,4,9,25,26,27,28,29,30] have documented varying conclusions regarding the scaling of facial asymmetry and the threshold of recognition. We opted to employ the three most prevalent criteria for evaluating facial asymmetry recognition rates via mirroring overlapping analysis.

According to the first criterion, facial asymmetry was classified according to Root Mean Squared Error (RMSE):

0 ≤ RMSE < 0.5 mm, mild asymmetry;

0.5 mm ≤ RMSE < 1 mm, moderate asymmetry;

RMSE ≥ 1mm, severe asymmetry.

According to the second criterion, facial asymmetry was classified according to Maximum Facial Misalignment (MFM):

0 ≤ MFM < 3 mm, mild asymmetry;

3 mm ≤ MFM < 6 mm, moderate asymmetry;

MFM ≥ 6 mm, severe asymmetry.

According to the third criterion, facial asymmetry was also classified according to Maximum Facial Misalignment (MFM):

0 ≤ MFM < 1 mm, mild asymmetry;

1 mm ≤ MFM < 2 mm, moderate asymmetry;

MFM ≥ 2 mm, severe asymmetry.

As for the wireframe template, the criterion of facial asymmetry was established as follows:

0 ≤ AAI < 1%, mild asymmetry;

1% ≤ AAI < 3%, moderate asymmetry;

AAI ≥ 3%, severe asymmetry.

As for the definition of asymmetry in five regions of each patient, when all parameters in the X, Y, and Z axis are recognized as “mild”, the patient is defined with mild asymmetry. If one of these parameters is recognized as others, moderate or severe asymmetry is defined. And, by comparing recognition rates of moderate and severe asymmetry of the two methods, the overall consistency rates of patients were calculated. Similarly, as for the five regions across three dimensions, the consistency rates were calculated by directly comparing recognition rates of moderate and severe asymmetry of the two methods.

Consistency rates of the mirroring and overlapping analysis and the wireframe template analysis were calculated as follows. If the recognition rates of the mirroring and overlapping analysis (Recognition rates of mirroring) are lower than the recognition rates of the wireframe template analysis (Recognition rates of the template), consistency rates were calculated with the following formula:

Consistency rates = Recognition rates of mirroring/Recognition rates of template × 100%. (2)

If not, consistency rates were calculated with the following formula:

Consistency rates = Recognition rates of template/Recognition rates of mirroring × 100%.(3)

### 2.4. Statistical Analysis

Measurements from the pre-experiment by the wireframe template analysis were performed by a single author and repeated once under the same conditions after one week. The two sets of data were statistically analyzed using the KAPPA analysis by SPSS software (version 24.0, IBM Corporation, Armonk, NY, USA).

## 3. Results

### 3.1. Kappa Value of the Pre-Experimental Wireframe Template Analysis

Kappa analysis of the first and the repeated measurements of the same pre-experimental samples by the wireframe template analysis was conducted. The results are shown in Table 2.

### 3.2. Overall Recognition Rates of the Mirroring and Overlapping Analysis and the Wireframe Template Analysis of Asymmetric Patients

The asymmetry recognition rates of the mirroring and overlapping analysis were evaluated with three different criteria. According to the first criterion, there were 16, 22, 16, 18, and 19 patients with moderate-to-severe asymmetry in the labial, mandibular angle, cheek, chin, and articular regions, respectively. According to the second criterion, there were 17, 21, 16, 18, and 24 patients with moderate-to-severe asymmetry in the labial, mandibular angle, cheek, chin, and articular regions, respectively. According to the third criterion, there were 21, 23, 21, 22, and 24 patients with moderate-to-severe asymmetry in the labial, mandibular angle, cheek, chin, and articular regions, respectively.

The results of the wireframe template analysis were that all 24 patients had moderate-to-severe asymmetry in the labial, mandibular angle, cheek, chin, and articular regions (Table 3).

### 3.3. Three-Dimensional Recognition Rates of the Mirroring and Overlapping Analysis and the Wireframe Template Analysis of Asymmetric Patients

Three-dimensional recognition rates of the mirroring and overlapping analysis and the wireframe template analysis of asymmetric patients in five regions of interest are shown in Table 4. Recognition rates of the mirroring and overlapping analysis became higher when using the second criterion and the highest with the third criterion.

### 3.4. Consistency Test of Results of the Mirroring and Overlapping Analysis and the Wireframe Template Analysis

#### 3.4.1. Overall Consistency Rates of Asymmetry in Five Regions of Interest

According to the first criterion, the mirroring and overlapping method identified 16, 22, 16, 20, and 19 patients with moderate-to-severe asymmetry in the labial, mandibular angle, cheek, chin, and articular regions, respectively, whereas the wireframe template identified 24 patients no matter where were measured, with consistency rates of 66.7%, 91.7%, 66.7%, 83.3%, and 79.2%, respectively, with an overall average consistency rate of 75.86%.

Similarly, according to the second criterion, the mirroring and overlapping method identified 17, 21, 16, 18, and 24 patients with moderate-to-severe asymmetry in the labial, mandibular angle, cheek, chin, and articular regions, respectively, whereas the wireframe template identified 24 patients no matter where were measured, with consistency rates of 70.8%, 87.5%, 66.7%, 75%, and 100%, respectively, with an overall average consistency rate of 80%.

Finally, according to the third criterion, the mirroring and overlapping method identified 21, 23, 21, 22, and 24 patients with moderate-to-severe asymmetry in the labial, mandibular angle, cheek, chin, and articular regions, respectively, whereas the wireframe template identified 24 patients no matter where were measured, with consistency rates of 87.5%, 95.8%, 87.5%, 91.7%, and 100%, respectively, and the overall average consistency rate was 92.5%. These results were documented in Table 5 specifically.

If the consistency rate is more than 85%, the consistency is considered to be good. Consistency rates of the third criterion in all regions were more than 85%, and the criterion was found suitable to evaluate the consistency rates of the two methods.

#### 3.4.2. Three-Dimensional Consistency Rates of Asymmetry in Five Regions of Interest

In the same way, to calculate the overall consistency rates, three-dimensional consistency rates of asymmetry in five regions of interest were calculated according to the first, second, and third criterion, respectively, and shown in Table 6.

According to the first criterion, regions with consistency rates of more than 85% were the *x*-axis of the mandibular angle, chin, and articular region. According to the second criterion, regions with consistency rates of more than 85% were the *x*-axis of the mandibular angle, chin, articular region, and the *y*-axis of the chin region. According to the third criterion, regions with consistency rates of more than 85% were the *x*-axis and the *z*-axis of all regions, and the *y*-axis of the mandibular angle, chin, and articular region. Whatever the criterion, the consistency rate of the *y*-axis of the cheek region was relatively low compared with others.

The third criterion was also found suitable to evaluate the consistency rates of the two methods in three dimensions.

## 4. Discussion

The increasing focus on aesthetic refinement has brought about an enhanced awareness of facial asymmetry, which has a potential disruption to the overall facial aesthetic and negative effects on the psychological well-being of individuals. Facial asymmetry arises from various factors, including the three-dimensional aspects of both soft and hard tissues in horizontal, vertical, and sagittal planes. O’Grady KF et al. [31] have tested six methods of evaluating facial asymmetry and evaluated asymmetry not only in one dimension but in x, y, and z dimensions. Asymmetry occurs due to the intricate three-dimensional variations in both soft and hard tissues.

Given the increasing use and acceptance of 3D technology, the importance of using 3D evaluation techniques to identify facial asymmetry and its usefulness in clinical diagnosis and treatment is increasingly recognized [32,33,34,35]. However, compared to the CBCT assessment of the craniofacial bone and the conventional 2D photographic evaluation, there is currently no consistent standard for evaluating soft tissues in three dimensions. Consequently, additional research is required to develop a more comprehensive and user-friendly approach to assessing asymmetry that encompasses three-dimensional irregularities in soft tissues. Such an approach will assist orthodontists in identifying soft tissue facial asymmetry of different regions in three dimensions.

For 3D diagnostic applications, the innovation of assessing human aesthetics through morphological analysis has garnered substantial attention among researchers. Standardized human facial templates were employed to evaluate the aesthetics of facial features, such as the nose and labia [36,37]. However, templates derived from averaged values tend to overlook individual and racial variations, leading to inherent limitations in their applicability. Therefore, these methodologies have not gained widespread acceptance clinically.

Furthermore, the limited number of landmarks used in visualizing facial features has left room for improvement in the technical sensitivity of clinical applications. Hajeer et al. [38] have established a landmark-based method to evaluate facial asymmetry in three dimensions by using a total of 19 facial landmarks—endocanthion, exocanthion, nasion, pronasale, alar curvature, subnasale, cheilion landmarks, superior labial sulcus, stomion superius, stomion inferius, labiale superius, labiale inferius, inferior labial sulcus, pogonion, and menton but the measurement indicators are not so much and have limitations. Verhoeven T [39] and Alqattan M [40] et al. find that the results for the asymmetry of facial landmarks have to be interpreted with regard to their reliability of identification. Considering the increasing prevalence of digital technology, the integration of artificial intelligence-assisted landmark identification holds the potential to enhance the effectiveness of 3D soft tissue evaluation techniques reliant on landmarks. The development of a more precise artificial intelligence-based landmark methodology is poised to reduce barriers to entry for clinical applications [41].

Therefore, we established a new method—a three-dimensional soft tissue spatial angle wireframe template to solve these problems. By using the wireframe template, clinicians can not only tell the accurate areas of the facial asymmetry but also show its direction in three dimensions. Furthermore, it will help the design of the treatment of facial asymmetry because we can know the specific area where asymmetry exists. Despite these, it can be used to evaluate the treatment effect of all asymmetrical patients and the growth and development of young patients.

This study aimed to test the accuracy and efficacy of the wireframe template further and guide the work on the perfection of the new method. According to Table 2, except for the *z*-axis of the articular region, the kappa values of other axes of the five regions are all more than 0.8, which represents the two measurements of them by the wireframe template method are almost perfectly consistent. As for the *z*-axis of the articular region, the consistency is also substantial.

A relatively strict criterion to evaluate facial asymmetry in a previous study [26] was used to perform a consistency test between the mirroring and overlapping analysis and the wireframe template analysis. The results indicated a slight deficiency in consistency, but the wireframe template retained a specific asymmetry recognition rate in different face regions. When we referred to other related studies [3], which adopted a more lenient threshold for recognizing facial asymmetry, a significant enhancement in the consistency between the two methods was observed.

The three most commonly used recognition criteria were selected. The first criterion is based on the RMSE, but it can only reflect an average scene of overall facial asymmetry and will hide the severe asymmetry we need to detect. As for the second criterion, it looks like the criterion agrees with our first sense. The wider threshold can make people focus on the more severe asymmetrical patients but ignore the unobvious asymmetry we can not easily find, which is important for clinicians to recognize.

According to Table 5 and Table 6, the third criterion was finally chosen both on overall and three-dimensional recognition, though it may recognize the asymmetry more sensitively, some tiny facial asymmetry that clinicians cannot recognize through their eyes can be detected by this method and more detailed messages on our face can be provided. Because mild asymmetry in certain facial regions is commonly observed in the general population, clinicians are susceptible to disregarding tiny facial asymmetry in their patients and may mistakenly conflate asymmetry in distinct regions of interest only through visual examination [42]. Therefore, the recognition of tiny facial asymmetry assumes paramount importance in providing guidance to clinicians [43].

Additionally, the sensitivity of the wireframe template for recognizing facial asymmetry is proven to be high. It also can facilitate the localization of asymmetrical regions and possess a distinct diagnostic impact on the unrecognizable asymmetry, which is valued a lot for diagnosis.

The *x*-axis asymmetry is always observed and considered as the main region of facial asymmetry. However, it is found asymmetry also exists in the *y*-axis and *z*-axis and plays important roles in the overall asymmetry of a region. It is suggested that facial asymmetry should not be diagnosed only by the front faces but in three dimensions in clinical practice.

During the measurement, it was also found that recognition rates of the asymmetry on the *y*-axis in the cheek regions are almost the least, no matter what criterion is chosen. It may suggest that asymmetry in the cheek regions may be predominantly in the horizontal and sagittal planes, with fewer cases in the *Y*-axis. The wireframe template analysis, known for its sensitivity to subtle asymmetries, may have identified more *Y*-axis asymmetries. And, the small sample size could also contribute to the results, which recommended us to study a larger scale of patients. Additionally, manual landmark positioning, despite re-measurements, could introduce errors. We are aiming to train AI for automatic landmarking to eliminate manual errors and achieve full automation of the 34 landmarks. Lastly, the low consistency rates in this area might highlight the need to reevaluate and integrate additional angular parameters to improve consistency.

In the mandibular angle regions and the articular regions, it was observed that the consistency rates of the two regions were relatively high. This could be attributed to the significant impact of the mandibular angle and articular regions on facial asymmetry, as indicated by previous studies [44,45,46,47], and the high sensitivity of the wireframe template in identifying these specific areas.

Furthermore, compared with the mirroring and overlapping method, the wireframe template is established by using the soft tissue landmarks that can suggest specific visualization information in three dimensions, in addition to its line spacing and angular data, which can help the clinicians distinguish the asymmetry present in the sagittal, vertical, and coronal directions of the patients. In this study, a novel method of evaluating facial asymmetry based on human morphology was preliminarily explored, with the traditional mirroring and overlapping method as a reference. The preliminary results of the comparison show that the wireframe template exhibited a great ability to recognize facial asymmetry and requires further improvement and research on the cheek regions.

The current use of artificial intelligence (AI) is a trend and direction for the future. Recent studies [48,49,50] found that AI-based cephalometric analysis was an effective tool and might offer a clinically acceptable diagnostic evaluation of facial asymmetry, enable prompt assessment, and reduce the effort involved in orthodontic diagnosis. Yurdakurban E [51] et al. used AI to explore the level of agreement between the conventional method and a machine-learning approach to facial asymmetry assessment on two-dimensional photographs and discovered that machine-learning algorithms had the potential for clinical use in asymmetry assessment and could help clinicians in a manual approach. These AI explorations are limited to two dimensions, but facial asymmetry is shown in the three-dimensional structure of the face.

Hidaka T [52] et al. developed an AI-designed real-time facial asymmetry analysis on dynamic facial asymmetry, but focusing only on oral displacement and eyebrow displacement for facial paralysis patients. Zhu Y [53] et al. developed a novel deep learning model to automatically construct a 3D symmetry reference plane, which was important to 3D facial symmetry analysis. Ho CT [54] et al. used AI to reconstruct Cone-beam computed tomography into 3D contour data and create a facial symmetry quantification system, they found AI helped to achieve rapid and accurate evaluations, facilitating better communication between clinicians and patients.

These studies explored the diagnosis of 3D facial asymmetry, but there is currently no systematic and detailed method for diagnosing and analyzing facial soft tissue asymmetry using AI. In the process of data analysis using the wireframe template analysis, AI was incorporated, but due to insufficient data and inadequate AI training, manual identification of some facial landmarks was still required. Although the results of the Kappa analysis were good, this still inevitably led to measurement errors. In the future, we will increase the sample size, train AI to be able to identify all landmarks without manual identification, construct the wireframe template, and determine the degree of asymmetry directly.

There are also many studies [55,56,57,58,59] researching the diagnosis of skeletal facial asymmetry deformities through CBCT. The joint analysis of soft and hard tissue asymmetry combined with CBCT data and facial scan data is a topic that urgently needs to be studied, and we will continue to explore the issue in the future.

## 5. Limitations

Due to the limited size of the sample, only a preliminary exploration of facial asymmetry recognition was conducted and the determination of accurate thresholds for evaluating facial asymmetry remains incomplete; therefore, it is imperative to augment the sample size to acquire more precise thresholds for assessing asymmetry in three dimensions. We will continue to explore the universality and effectiveness of the wireframe template analysis with a larger group of patients in the future.

Recent studies [60,61,62] found reliable differences in facial asymmetry between databases from different ethnicities. Considering that, we will also enrich our data sources while further increasing the sample size, analyze facial scan data from multiple ethnicities to explore the impact of racial differences in facial structure on the results of the study, and draw more convincing conclusions in the future.

Similarly, considering the precision of landmark positioning and low consistency rates of the *y*-axis of the cheek region, the landmarks and parameters still have room to improve. In the future, we will continue to improve and develop the template further, exploring the subjective and objective evaluation of facial asymmetry by using our wireframe template and applying it to help guide the diagnosis and treatment of facial asymmetry when making clinical decisions.

## 6. Conclusions

The accuracy and efficacy of the 3D facial wireframe template are tested by comparing it with the mirroring and overlapping method both on overall and three-dimensional diagnosis. The template will provide useful guidance for clinicians to find facial asymmetry of different regions in three dimensions and detect tiny facial asymmetry that people’s eyes cannot find easily.

## Figures and Tables

**Table 1:** Angular parameters for evaluating asymmetry for each site in three dimensions in the wireframe template.

Position of Asymmetry	Dimension of Asymmetry	Angular Parameters
Labial	X	∠Sal-Ch-Stb
	Y	∠Sal-Go-Ch
	Z	∠Sal-Ch-As
Mandibular Angle	X	∠Me-Go-Mt
	Y	∠Tr-Zg-Go
	Z	∠Zg-Tr-Go
Cheek	X	∠Ex-Zg-Sal
	Y	∠Zg-Tr-He
	Z	∠Tr-Zg-Ex
Chin	X	∠Gn-Mt-Me
	Y	∠Li-Mt-Gn
	Z	∠Me-Go-Mt
Articular	X	∠Ex-Tr-Go
	Y	∠Tr-Zg-Go
	Z	∠Tr-Go-Zg

**Table 2:** Kappa analysis of the first and the repeated measurements of the same pre-experimental samples by the wireframe template analysis.

Position of Asymmetry	Dimension of Asymmetry	Kappa Value
Labial	X	0.92
	Y	1
	Z	1
Mandibular Angle	X	1
	Y	0.89
	Z	0.91
Cheek	X	0.93
	Y	0.82
	Z	1
Chin	X	0.84
	Y	1
	Z	1
Articular	X	1
	Y	0.87
	Z	0.664

**Table 3:** Overall recognition rates of the mirroring and overlapping analysis and the wireframe template analysis of asymmetric patients in five regions of interest.

Method	Criterion	Overall Recognition Rates				
		Labial	Mandibular Angle	Cheek	Chin	Articular
The Mirroring and Overlapping Analysis	The First Criterion	16/24	22/24	16/24	18/24	19/24
	The Second Criterion	17/24	21/24	16/24	18/24	24/24
	The Third Criterion	21/24	23/24	21/24	22/24	24/24
The Wireframe Template Analysis		24/24	24/24	24/24	24/24	24/24

**Table 4:** Three-dimensional recognition rates of the mirroring and overlapping analysis, and the wireframe template analysis of asymmetric patients in five regions of interest.

Position of Asymmetry	Dimension of Asymmetry	Recognition Rates of The Mirroring and Overlapping Analysis			Recognition Rates of Wireframe Template
		The First Criterion	The Second Criterion	The Third Criterion	
Labial	X	14/24	14/24	19/24	21/24
	Y	3/24	14/24	19/24	23/24
	Z	11/24	16/24	21/24	24/24
Mandibular Angle	X	22/24	21/24	23/24	22/24
	Y	14/24	18/24	22/24	22/24
	Z	6/24	17/24	20/24	23/24
Cheek	X	7/24	10/24	20/24	19/24
	Y	3/24	7/24	13/24	22/24
	Z	16/24	14/24	20/24	19/24
Chin	X	20/24	18/24	22/24	20/24
	Y	7/24	18/24	22/24	21/24
	Z	11/24	17/24	20/24	22/24
Articular	X	19/24	22/24	24/24	21/24
	Y	3/24	17/24	21/24	22/24
	Z	5/24	16/24	20/24	21/24

**Table 5:** Overall consistency rates of the mirroring and overlapping analysis and the wireframe template analysis in five regions of interest.

Criterion	Overall Consistency Rates					Average Consistency Rates
	Labial	Mandibular Angle	Cheek	Chin	Articular	
The First Criterion	66.7%	91.7%	66.7%	83.3%	79.2%	75.86%
The Second Criterion	70.8%	87.5%	66.7%	75%	100%	80%
The Third Criterion	87.5%	95.8%	87.5%	91.7%	100%	92.5%

**Table 6:** Three-dimensional consistency rates of the mirroring and overlapping analysis and the wireframe template analysis in five regions of interest.

Position of Asymmetry	Dimension of Asymmetry	Consistency Rates of The First Criterion	Consistency Rates of The Second Criterion	Consistency Rates of The Third Criterion
Labial	X	66.7%	66.7%	90.5%
	Y	13.0%	60.9%	82.6%
	Z	45.8%	76.2%	87.5%
Mandibular Angle	X	100.0%	95.5%	95.7%
	Y	63.6%	81.8%	100.0%
	Z	26.1%	73.9%	87.0%
Cheek	X	36.8%	52.6%	95.0%
	Y	13.6%	31.8%	59.1%
	Z	84.2%	73.7%	95.0%
Chin	X	100.0%	90.0%	90.1%
	Y	33.3%	85.7%	95.7%
	Z	50.0%	77.3%	90.1%
Articular	X	90.5%	95.5%	87.5%
	Y	13.6%	77.3%	95.5%
	Z	23.8%	76.2%	95.2%

## Data Availability

Data available on request due to privacy and ethical problems. The data presented in this study are available on request from the corresponding author. The data are not publicly available due to the fact that the clinical data of all participants in this study belongs to Peking University School and Hospital of Stomatology and we need to obtain the approval of the hospital’s medical department when obtaining it.
